# Effects of exercise on physical function and pain in adults with rheumatoid arthritis: an exit meta-analysis of randomized controlled trials

**DOI:** 10.1007/s10067-026-08274-w

**Published:** 2026-07-08

**Authors:** George A. Kelley, Kristi S. Kelley, Kim M. Huffman

**Affiliations:** 1 School of Public and Population Health, Boise State University, Boise, ID 83725-1835 USA; 2 Division of Rheumatology & Immunology, Duke Molecular Physiology Institute, Human Physiology Testing Core, Duke University School of Medicine, Duke University, Durham, NC 27701 USA

**Keywords:** Exercise, Meta-analysis, Pain, Physical function, Rheumatoid arthritis

## Abstract

The most recent American College of Rheumatology Guidelines concluded, based on meta-analyses, that moderate certainty evidence from randomized controlled trials (RCT) existed to recommend that exercise, compared to no exercise, increased physical function and reduced pain in adults with rheumatoid arthritis (RA). However, effect size stability was not quantitatively established. To address this gap, the purpose of this study was to use quantitative methods to determine whether further RCT and meta-analyses are needed or stability has been reached. Using meta-analytic data from these guidelines, a recently developed and novel approach, the Doi–Abdulmajeed Trial Stability (DAts) index was used to determine effect size stability, i.e., exit status, of exercise on physical function and pain in adults with RA. A DAts < 0 = exit, 0–0.05 = possibly exit, and > 0.05 = no exit. Across all types of exercise, the DAts index was 0.0095 for physical function (20 studies, 1487 participants) and 0.0018 for pain (14 studies, 998 participants), suggesting possible exit status. These findings add to the American College of Rheumatology Guidelines assessment of “moderate” strength of evidence rating, suggesting that additional RCT and subsequent meta-analyses are needed on the effects of exercise on physical function and pain in adults with RA before true stability/exit status can be reached.

**Table:** 

**Key Points** • *A novel quantitative method was used to examine the stability, i.e., exit status, of exercise on physical function and pain in adults with rheumatoid arthritis (RA).* • *Only "possible exit" status was found for the eff ects of exercise on physical function and pain in adults with RA.* • *Additional studies are needed on the eff ects exercise on physical function and pain in adults with RA before stability can be determined.*

**Supplementary Information:**

The online version contains supplementary material available at https://doi.org/10.1007/s10067-026-08274-w.

## Introduction

Rheumatoid arthritis (RA) is a chronic, autoimmune disease that causes inflammation and damage to joints resulting in reduced physical function as well as increased pain [1]. More common in women than men [2], both the incidence and prevalence of RA are increasing [3, 4] as well as costly [5, 6]. For those with RA, physical function and pain are outcomes that patients deem as important [7]. Not surprisingly, the prevalence of reduced physical function as well as increased pain in those with RA is high [8–10], including when compared to the general population [11].

As part of a comprehensive approach in the treatment of RA, the 2022 American College of Rheumatology Guidelines concluded, based on the conduct of their own meta-analyses, that there was “moderate” certainty of evidence (collectively) that exercise (aerobic, aquatic, resistance, mind–body) improved physical function (19 studies) and reduced pain (14 studies) in adults with RA [12]. Consistent engagement in exercise was recommended for those with RA, and decisions regarding the level of certainty were based on using the Grading of Recommendations Assessment, Development and Evaluation (GRADE) framework [13].

There has been increased criticism regarding the conduct of redundant studies and meta-analyses on the same topic, often referred to as research waste [14, 15]. Research waste can lead to studies that have little scientific value as well as the misallocation of resources. To try and address this issue, Abdulmajeed et al. [16] have recently developed a novel approach known as the Doi–Abdulmajeed Trial Stability (DAts) index as well as a convergence plot to accompany it. This approach provides a quantitative assessment of the conclusiveness, i.e., exit status, of a meta-analysis, suggesting whether or not additional studies are needed [16]. More specifically, the DAts index and subsequent convergence plot are designed to (1) reduce Type 2 errors by providing a quantitative measure of stability, helping to identify when a meta-analysis is conclusive, and thus, reducing the risk of missing a real effect (false negative/Type 2 error); (2) act as a “stop” signal where the DAts score assists in defining when a meta-analysis is considered “conclusive,” and thus, serving as a threshold to avoid unnecessary further studies; and (3) show effectiveness. Thus, this method could contribute to guidelines development, evidence surveillance and research prioritization. Using this approach, the current study examined the exit status regarding the overall effects of exercise on physical function and pain based on meta-analytic exercise data from RCTs reported in the 2022 American College of Rheumatology Guidelines [12].

## Methods

### Overview

Where appropriate, this exit meta-analysis followed the Preferred Reporting Items for Systematic Reviews and Meta-Analyses (PRISMA 2020) Statement [17]. The a priori protocol for this study was registered in Open Science Framework on November 16, 2025 (see https://osf.io/3j2a8/overview). It is noted here that the focus of this study was on the original intent of meta-analysis, that is, to reach general conclusions regarding a body of research [18]. In addition, and similar to most guidelines, this was the intent of the original 2022 American College of Rheumatology Guidelines [12] from which the data for this study were drawn. Institutional review board and ethics committee approval was not needed for this study since it was based on summarized, de-identified data that is freely available and previously published in a peer-reviewed journal [12]. No artificial intelligence was used for this study.

### Data source

Data were derived from the 2022 American College of Rheumatology Guidelines [12] in which meta-analyses were restricted to the following as described on page 339 of the Supplementary file of the original article: (1) randomized trials only, (2) comparisons to no exercise group, (3) pain and function outcomes, and (4) direct measurements (no surrogates) ≥ 12 weeks (their threshold for long-term) [12]. Exercise group interventions as defined in the original guidelines article included aerobic, aquatic, resistance, and mind–body activities for physical function (19 studies, 1487 participants) [19–37] and/or pain (14 studies, 998 participants) [19, 21, 23, 25–27, 29, 30, 32–35, 38, 39] in adults with RA [12]. No exercise group comparators included either usual care in which participants were instructed to maintain their usual activities, including pharmacologic treatment regimen [19, 20, 22, 30, 32, 34, 35, 38, 39], educational advice on RA that included information on physical activity but no physical intervention [23, 24, 28, 29, 33, 36]; sham intervention that consisted of range of motion exercises [25, 26, 31]; drug treatment for RA that included the same drug treatment as the exercise group [21, 37]; or progressive relaxation [27]. Additional details regarding individual studies can be found in the original guidelines report [12]. The authors focused on these recent guidelines versus original trials or stand-alone meta-analyses because clinicians and others increasingly rely on objective third parties such as the American College of Rheumatology to aid in their decision-making [12].

### Data abstraction

Using Microsoft Excel workbooks, the first and second authors conducted dual, independent data extraction for data reported for the RCT exercise studies in the guidelines statement [12]. Data extracted from each study included author names, the year the study was published, as well as sample sizes, means, standard deviations, mean treatment effects, and 95% CI for treatment effects for physical function and pain. The authors then met to discuss their selections. Any disagreements were resolved through discussion. If agreement was not reached, the third author provided a final decision. Prior to meeting, Gwet’s AC_1_ statistic was used to assess inter-rater agreement [40].

### Research synthesis

#### Effect size

The primary outcomes were physical function and pain. The standardized mean difference (SMD) effect size (Hedge’s *g*) was the metric of choice for both physical function and pain given that different studies measured these outcomes differently. This was similar to the approach used in the guidelines statement [12].

#### Aggregate data treatment effects meta-analysis

Prior to conducting stability, i.e., exit meta-analyses, a traditional meta-analysis was conducted using the inverse variance heterogeneity (IVhet) model of Doi et al. [41] versus the random-effects model of Dersimonian and Laird [42] used in the original meta-analyses [12]. The IVhet model was chosen over others because it has been shown to provide better coverage of 95% CI than the random-effects models currently available [41, 43–45]. Non-overlapping 95% confidence intervals (CI) as well as two-tailed alpha (*p*) values ≤ 0.05 based on the *z*-distribution were considered statistically significant. Consistent with the original guidelines report, the minimum detectable change (MDC) for both physical function and pain was set at a SMD of 0.15 [12].

Heterogeneity was examined using the *Q* statistic with an alpha (*p*) value ≤ 0.10 considered to represent statistically significant heterogeneity, consistent with current practice [46, 47]. In addition, inconsistency was examined using the *I*^2^ statistic, including 95% CI for *I*^2^ [48]. Furthermore, analyses extended beyond those conducted in the guidelines statement [12] to include (1) 95% prediction intervals (PI) to estimate absolute between-study heterogeneity, and thus, what effect one might expect if one conducted their own RCT in similar populations [49]; (2) leave-one-out analysis to determine what effect each included study had on the overall pooled findings; (3) outlier analysis by deleting those studies in which any part of their point estimates and 95% CI was not included in the overall pooled estimate; (4) number needed-to-treat (NNT), derived from a distribution-based approach for SMD effect sizes [50]; (5) worldwide gross estimates of the number of RA patients who could improve their physical function and pain by exercising, derived from the prevalence of those with RA who report no regular physical activity (79%) [51] and the number of people worldwide with RA (17.9 million) [3]; and (6) instrument to assess the Credibility of Effect Modification Analyses (ICEMAN) based on subgroup analyses according to type of exercise (aerobic, resistance, aquatic, mind–body) as reported in the original guidelines statement [12]. Based on up to nine possible questions, overall credibility is categorized as either “very low”, “low”, “moderate”, or “high” [52]. If rated as “very low” credibility, it is recommended that the focus be on overall versus subgroup effects [52]. Per ICEMAN recommendations, such assessments were only conducted if a statistically significant between-group (*Q*_b_) interaction was found given that the purpose of ICEMAN is to examine the assertion of effect modification rather than a conclusion of no effect modification [52]. The authors also note that the 95% PI in the current study uses the *t*-distribution to address uncertainty in tau-squared as well as incorporate the standard error for uncertainty in the mean [49].

Small-study effects, of which publication bias is one possibility [53], were examined using the Doi plot and LFK index, the former of which has been suggested to be more intuitive than the funnel plot, and the latter more robust than Egger’s regression-intercept test, especially when the number of effect sizes is small [54]. Values for the LFK index within ± 1 represent no symmetry, greater than ± 1 but within ± 2 minor asymmetry, and greater than ± 2 major asymmetry [54].

Based on the suggestion of one of the reviewers for the current study, a post hoc decision was made to conduct additional moderator analyses beyond the type of exercise. This included moderator analyses according to type of control group, overall risk of bias, type of control group partitioned according to type of exercise (aerobic, resistance, aquatic, mind–body), as well as overall risk of bias according to type of exercise. If applicable, ICEMAN criteria were also used for any statistically significant moderator analyses.

In the guidelines article, from which the data for the current study was derived [12], the Cochrane Risk of Bias Instrument for randomized controlled trials was used, but no overall risk of bias was reported. Therefore, the first author requested necessary data from the corresponding author and American College of Rheumatology office on April 30 and May 8, 2026, respectively, receiving selected data on May 14, 2026. For the data provided, results were assessed as either “low”, “high”, or “unclear” risk of bias across six defined domains: (1) random sequence generation (selection bias), (2) allocation concealment (selection bias), (3) blinding of participants and personnel (performance bias), (4) blinding of outcome assessment (detection bias), (5) incomplete outcome data (attrition bias), and (6) selective reporting (reporting bias). However, to coincide with the more recent Cochrane risk of bias instrument for randomized controlled trials (RoB2) [55], the current authors assessed risk of bias as either “low” risk of bias, “high” risk of bias, or “some concerns” across five defined domains: (1) bias arising from the randomization process, (2) bias due to deviations from intended intervention, (3) bias due to missing outcome data, (4) bias in measurement of the outcome, and (5) bias in selection of the reported result [55]. Domains 1 and 2 in the original results were collapsed into domain 1 of the current assessment, with the lower of the two assessments chosen if differences existed. Assessment was conducted using the standardized Excel tool for RoB2 (version 2.0, available from www.riskofbias.info) while a traffic plot figure was generated using the robvis shiny app [56].

### Exit (stability) meta-analysis

For the primary purpose of this study, separate exit meta-analyses were conducted for physical function and pain in adults with RA, details of which have been described elsewhere [16]. Briefly, cumulative meta-analysis, ranked by year, was calculated followed by a final meta-analytic effect size using all available studies [16]. The Δ C index (difference between final meta-analytic result and cumulative meta-analysis at each step) was then used to calculate the DAts [16]. To assess stability, the DAts index acts as a threshold to determine if the effect size has stabilized [16]. Thus, it evaluates the convergence of the cumulative meta-analysis. A convergence plot is then produced to visualize when the DAts index has reached stability, i.e., exit status [16]. Stability, i.e., exit status, is achieved when the plot line flattens out after the accumulation of 50% of participants and the DAts index approaches zero for continuous data [16]. Thus, the flatter the horizontal line after accumulation of 50% of participants, the more likely that exit status has been reached, while a completely flat line indicates that exit status has been reached [16]. This is considered to indicate sufficient evidence, i.e., stability, and that further studies are unlikely to change the conclusion. If the plot line continues to fluctuate significantly and the DAts index remains high, this suggests that additional studies are needed before stability, i.e., exit status, can be reached [16]. A DAts value < 0 implies exit, i.e., stability status, between 0 and 0.05 possibly exit, and > 0.05 no exit [16]. Finally, specific numerical probabilities for Type 1 and Type 2 errors for the DAts index are not fixed at a single value for all scenarios as they depend on data stability. However, there is a parallel between Type I and II errors with sensitivity (Se) and specificity (Sp), that is, a Type I error is 1-Sp, i.e., false positive rate, and a Type II error is 1-Se, i.e., false negative rate. Thus, based on the data reported in the original Abdulmajeed et al.’s [16] study, the Type I error would be ~ 0.09 and Type II error ~ 0.13, both of which the investigative team believes is acceptable. Stata code for the DAts index is shown in Supplementary file 1.

For both physical function and pain, analyses included exit meta-analyses for pooled changes across all types of exercise. In addition, a post hoc decision was made to conduct additional exit meta-analyses based on the suggestion of one of the reviewers. This included separate exit meta-analyses according to type of exercise, type of control group, risk of bias, type of exercise partitioned according to type of control group, as well as type of exercise partitioned according to risk of bias. No separate exit meta-analysis was planned a priori for mind–body exercise since only one study [33] was included for both pain and physical function [12].

With oversight from the second and third authors, all statistical analyses were conducted by the first author using Stata (version 16.1) and interpreted by all authors. Any disagreements regarding the results and interpretation of such were discussed until mutual agreement was reached.

## Results

### Study characteristics

Data from all RCTs that examined the effects of ≥ 12 weeks of exercise (aerobic, aquatic, resistance, mind–body) compared to no exercise on self-reported or performance-based physical function (19 studies, 1487 participants) [19–37] and pain (14 studies, 998 participants) [19, 21, 23, 25–27, 29, 30, 32–35, 38, 39] from the 2022 American College of Rheumatology Guidelines [12] were included. Based on Gwet’s AC_1_ statistic, the overall agreement rate for data abstraction was 0.98 (95% CI, 0.96, 0.99). The number of effect sizes (*N* = 20) exceeded the number of studies (*N* = 19) for physical function because the Siqueira et al.’s [35] study included both a resistance training group and an aquatic exercise group. Ten studies included aerobic exercise [20–24, 28, 29, 36, 37, 39], six resistance exercises [25, 26, 30–32, 34], four aquatic exercises [19, 27, 35, 38], and one mind–body exercise [33].

Item, study-level, and overall risk of bias results are shown in Supplementary file 2. As shown, overall risk of bias was considered to be “high” for 14 of 21 studies (66.7%) [19, 21–23, 25, 27, 30, 31, 33–35, 37–39] and “some concerns” for 7 of 21 (33.3%) studies [20, 24, 26, 28, 29, 32, 36]. No studies were considered to have a “low” overall risk of bias.

### Treatment effects meta-analysis

#### Physical function

**Fig. 1:**
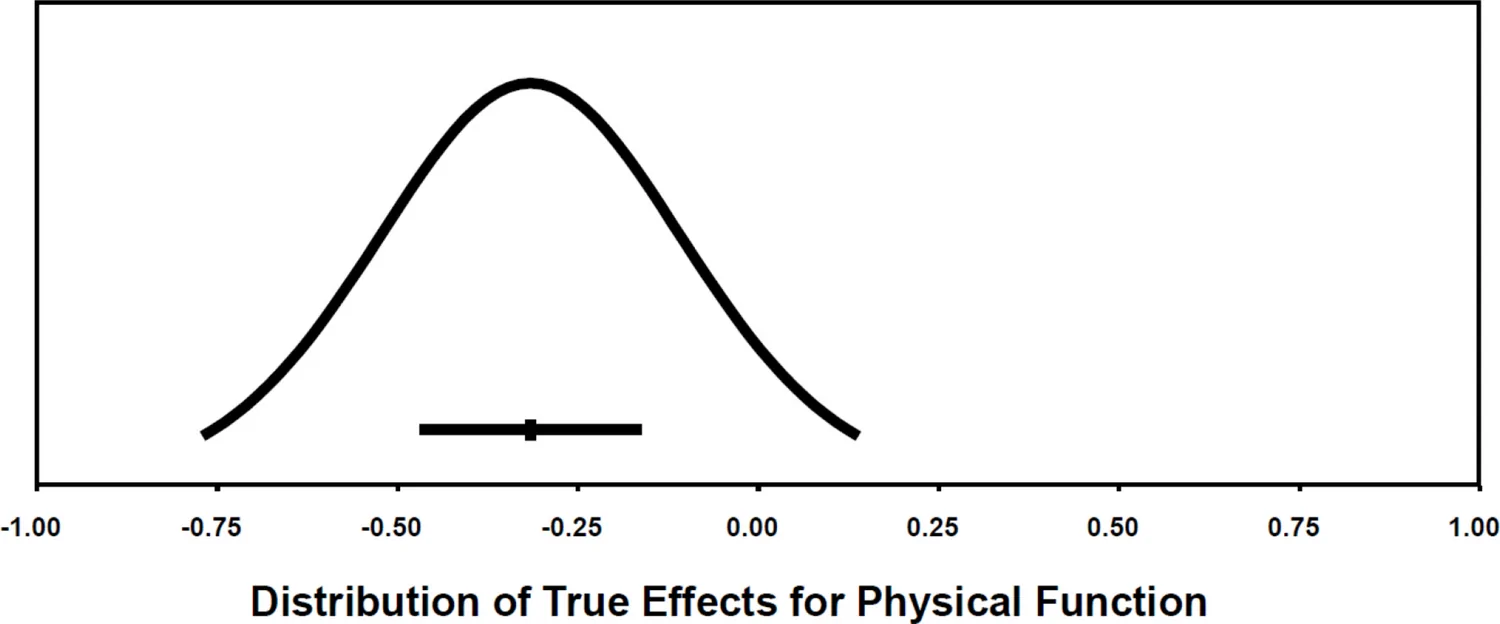
Prediction interval plot for changes in physical function. The left and right sides of the solid horizontal line represent the pooled lower and 95% confidence intervals (− 0.47 to − 0.16) while the black square represents the pooled effect size (Hedge’s *g*) for changes in physical function (− 0.32). The distribution curve represents the true effect size (pooled) in 95% of comparable populations (− 0.77 to 0.14)

Overall treatment effect results for physical function are shown in Table 1 while study and group-level results according to type of exercise are shown in Supplementary file 3. Given the lack of between-group differences (*Q*_b_ = 3.52, *p* = 0.32), results were pooled across all groups, and the ICEMAN criteria were not applied [12, 52]. At the individual study level, changes in physical function ranged from a SMD (Hedge’s *g*) of − 0.94 (95% CI, − 1.35 to − 0.53) to 0.38 (95% CI, − 0.37 to 1.13). When pooled, statistically significant improvements were observed with both the mean and 95% CI exceeding the MDC of 0.15. Based on the *Q* statistic, statistically significant heterogeneity was observed while the 95% CI for inconsistency based on *I*^2^ was wide. Tau-squared, an absolute measure of between-study heterogeneity, was 0.04 while tau was 0.20 (95% CI, 0.04 to 0.33). The 95% PI included the null, although most of the interval was on the side of benefit. Comparison of the mean and 95% CI with the 95% PI is shown in Fig. 1. As can be seen, the 95% PI was wider than the 95% CI, the former reflecting what results one might expect if they conducted their own study in comparable populations of those with RA. Minor asymmetry (LFK index =  − 1.33), suggestive of small-study effects was observed (Supplementary file 4). With each study deleted from the model once, including one outlier [21], results remained statistically significant, ranging from − 0.27 (95% CI, − 0.40 to − 0.14) to − 0.34 (95% CI, − 0.51 to − 0.18) (Supplementary file 5).

**Table 1:** Overall treatment effect changes based on SMD for physical function and pain

Variable	ES (#)	*N* (#)	$\overline{X }$(95% CI)^a^	*z* (*p*)	*Q* (*p*)	*I*^2^ (95% CI)	95% PI
Physical function	20	1487	− 0.32 (− 0.47, − 0.16)	− 4.05 (< 0.001)*	32.39 (0.03)**	41.3% (0, 66.8)	− 0.77, 0.14
Pain	14	998	− 0.33 (− 0.49, − 0.16)	− 3.85 (< 0.001)*	19.29 (0.11)	32.6% (0, 64.9)	− 0.75, 0.09

*SMD* standardized mean difference, *ES* effect sizes, *N* total number of participants, *Q* Cochrane *Q* statistic, CI confidence intervals, *PI* prediction intervals

^*^p ≤ 0.05 (statistically significant); **p ≤ 0.10 (statistically significant)

^a^Negative values are indicative of improvements associated with exercise

Based on the mean and 95% CI, the NNT was 6 (95% CI, 4 to 11). In terms of those with RA who reported no physical activity, it was estimated that 2,543,345 (95% CI, 1,272,817 to 3,672,987) worldwide could improve their physical function by exercising. When estimated based on the 95% PI, the NNT was more varied, ranging from 2 for improvements in physical function to 13 for no change or poorer physical function. Based on the 95% PI, it was estimated that up to 7,707,500 physically inactive adults with RA globally could improve their physical function by exercising while 1,103,042 could see no change or poorer physical function. The net benefit was 5,967,458 in favor of improvement (ratio = 6.41:1.00 in favor of exercise).

Moderator analyses are shown in Supplementary file 6. As can be seen, a statistically significant between-group difference was found for aquatic exercise between usual exercise and other types of controls. Based on ICEMAN criteria, the credibility of the observed difference was considered “very low”. No other statistically significant between-group differences were observed.

#### Pain

**Fig. 2:**
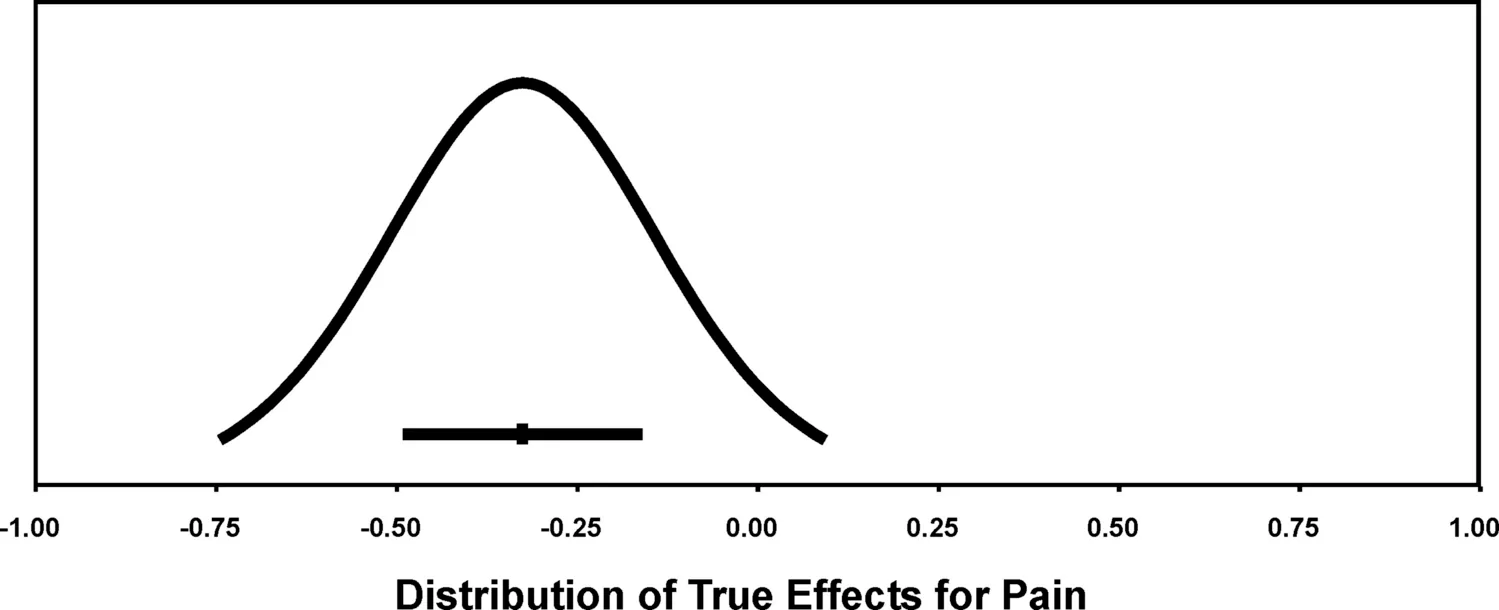
Prediction interval plot for treatment effect changes in pain. The left and right sides of the solid horizontal line represent the pooled lower and 95% confidence intervals (− 0.49 to − 0.16) while the black square represents the mean effect size (Hedge’s *g*) for changes in physical function (− 0.33). The distribution curve represents the true effect size (pooled) in 95% of comparable populations (− 0.74 to 0.09)

Overall treatment effect results for pain are shown in Table 1 while study and group-level results according to type of exercise are shown in Supplementary file 7. Because of the lack of between-group differences (*Q*_b_ = 0.17, *p* = 0.98), results were pooled across all groups, and the ICEMAN criteria were not applied [12, 52]. At the individual study level changes in pain ranged from a SMD (Hedge’s *g*) of − 0.93 (95% CI, − 1.69 to − 0.17) to 0.01 (95% CI, − 0.46 to 0.48). When pooled, statistically significant improvements similar to those for physical function were observed with both the mean and 95% CI exceeding the MDC of 0.15. No statistically significant heterogeneity based on the *Q* statistic was observed. However, the 95% CI for inconsistency based on *I*^2^ was wide. Tau-squared was 0.03 while tau was 0.17 (95% CI, 0.0 to 0.17). The 95% PI included the null, although most of the interval was on the side of reducing pain as a result of exercise. A comparison of the mean and 95% CI with the 95% PI is shown in Fig. 2. As shown, the 95% PI was wider than the 95% CI, with the 95% PI indicating what results one may expect to see if they conducted their own study in comparable RA populations. No asymmetry (LFK index =  − 0.98) suggestive of small-study effects was observed (Supplementary file 8). With each study deleted from the model once, results remained statistically significant, ranging from − 0.28 (95% CI, − 0.43 to − 0.12) to − 0.38 (− 0.55 to − 0.21) (Supplementary file 9).

Derived from the mean and 95% CI, the NNT for pain was 5 (95% CI, 4 to 11). In terms of those with RA who report no physical activity, it was estimated that 2,604,236 (95% CI, 1,272,817 to 3,842,663) worldwide could reduce their pain by exercising. When estimated based on the 95% PI, the NNT was more varied, ranging from 2 for improvements in physical function to 20 for no change or poorer pain. Based on the 95% PI, it was estimated that 5,702,016 physically inactive adults with RA globally could reduce their pain by exercising while 707,050 could see no change or increase in pain. The net benefit for decreases in pain was 4,994,966 people (ratio of 8.06:1.00 in favor of exercise).

Moderator analyses are shown in Supplementary file 10. As can be seen, no statistically significant between-group differences were observed.

### Exit meta-analysis

#### Physical function

**Fig. 3:**
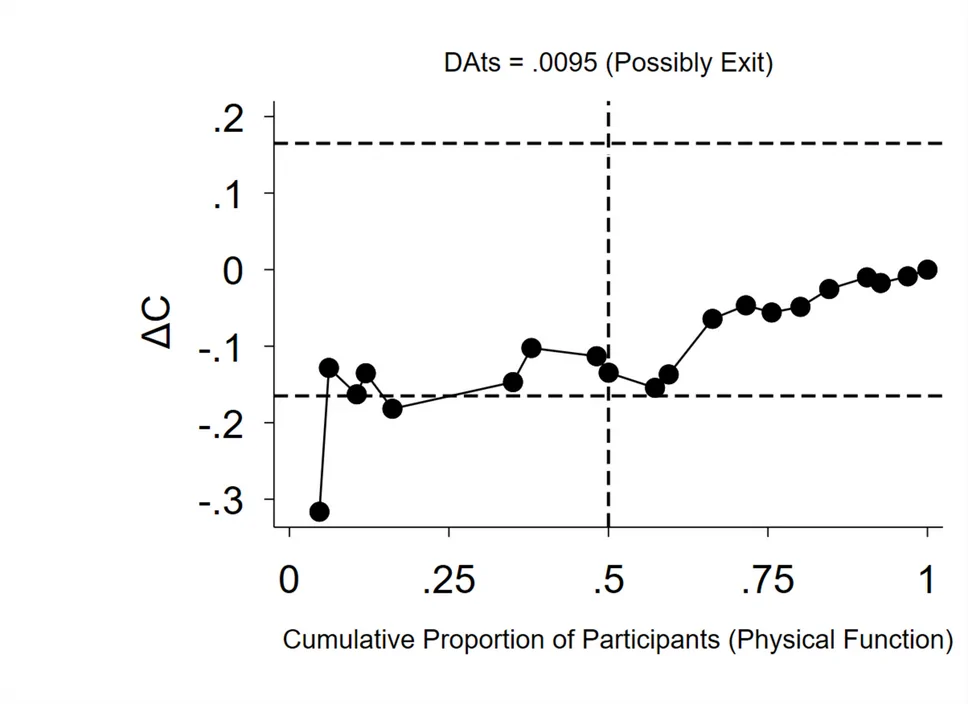
Doi-Abdulmajeed trial stability index (DAts) to determine the stability, i.e., exit status, for changes in physical function. The horizontal axis represents the cumulative proportion of RA patients from each study while the vertical axis represents the difference between the final meta-analytic result and the cumulative meta-analysis effect size at each step. Here the results flatten out somewhat after the accrual of 50% of participants but not enough to reach full exit status, suggesting the need for additional research on exercise and physical function in adults with RA before stability can be confirmed. For exit status, both a DAts below zero AND a delta C within +/− 0.15 are needed

For the primary purpose of this study, exit meta-analysis results for the stability of exercise and changes in physical function are shown in Fig. 3. As can be seen, the results flatten out somewhat after the accrual of 50% of participants but not enough to reach full exit status. The DAts index was 0.0095, suggesting only possible exit status. These findings suggest the need for additional research on exercise and physical function in adults with RA before stability can be confirmed, as the inclusion of such in future meta-analyses may have a significant impact on the estimated effect size, including the direction of effect.

Exit meta-analysis results for physical function according to subgroups are shown in Supplementary file 6 while results with each study deleted from the model once are shown in Supplementary file 11. For those groups in which a sufficient number of effect sizes were available, six, five, and four groups suggested either “exit”, “possibly exit”, or “no exit” status. With each study deleted from the model once, all results except one suggested “possible exit” status, consistent with overall pooled results.

### Pain

**Fig. 4:**
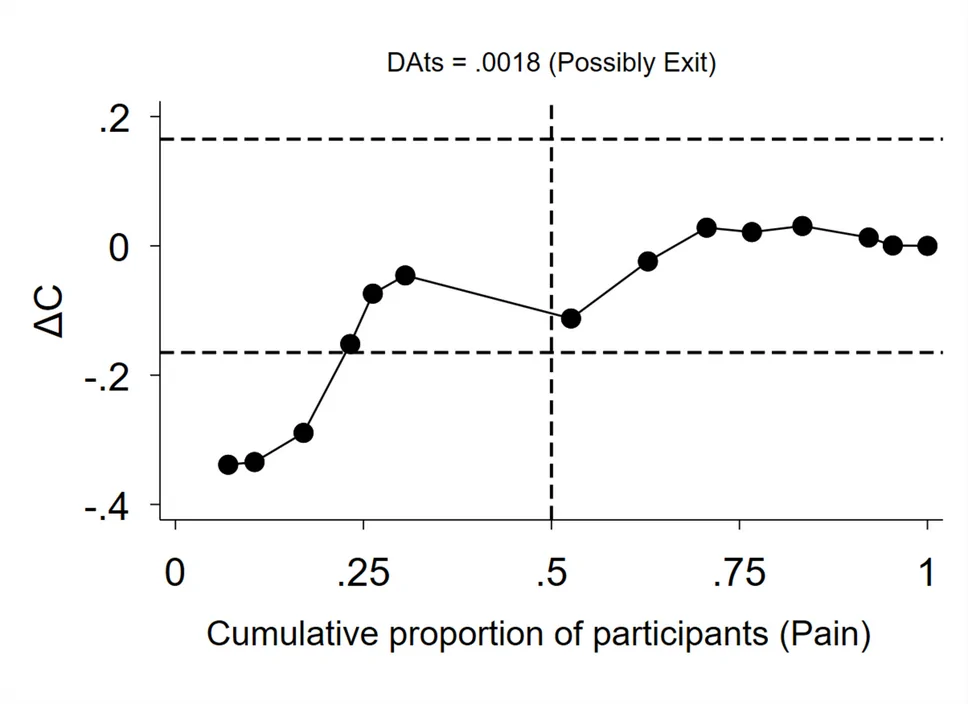
Doi-Abdulmajeed trial stability index (DAts) to determine the stability, i.e., exit status, for changes in pain. The horizontal axis represents the cumulative proportion of RA patients from each study while the vertical axis represents the difference between the final meta-analytic result and the cumulative meta-analysis effect size at each step. Here the results flatten out somewhat after the accrual of 50% of participants but not enough to reach full exit status, suggesting the need for additional research on exercise and physical function in adults with RA before stability can be confirmed. For exit status, both a DAts below zero AND a delta C within +/− 0.15 are needed

Exit meta-analysis results for the stability of exercise and changes in pain are shown in Fig. 4. Similar to physical function, the results flatten out somewhat after the accrual of 50% of participants but not enough to reach full exit status. The DAts index was 0.0018, suggesting only possible exit status. Thus, these findings suggest the need for additional research on exercise and pain in adults with RA before stability can be confirmed, as the inclusion of such in future meta-analyses may have a significant impact on the estimated effect size, including the direction of effect.

Exit meta-analysis results for pain according to subgroups are shown in Supplementary file 10, while leave-one-out analyses are shown in Supplementary file 12. For those groups in which a sufficient number of effect sizes were available, seven, four, and three groups suggested either “exit”, “possibly exit”, or “no exit” status. With each study deleted from the model once, half of the results suggested “possibly exit” while the other half suggested “exit”.

## Discussion

### Overall findings

With a focus on the original purpose of meta-analysis, that is, to reach general conclusions about a body of research [18], as well as the intent of the original guidelines from which data for this study were derived [12], the primary purpose of the current study was to examine the exit status, i.e., stability, of exercise on pain and physical function in adults with RA. Overall, the results of this first-ever study resulted in “possibly exit” status for both physical function and pain in adults with RA. These findings suggest that additional RCTs and meta-analyses based on RCT are needed before true stability can be established. Such meta-analyses may be conducted either separately or as part of future updated guidelines in the treatment of RA. From the investigative team’s perspective, these findings are important because they suggest that resources aimed at RCTs with respect to the overall effects of exercise on physical function and pain in adults with RA are still needed and will not be wasted by doing so. These findings add to the original guideline’s GRADE rating of “moderate” for both physical function and pain [12], that is, “the effects observed are likely to be close to the true estimate of effect but there is a possibility that it is substantially different” [57]. The similar results observed are notable given that both the DAts and GRADE assess different elements, and thus, could be considered complementary to each other [16]. However, if the results between the DAts and GRADE had been substantially different, one would have needed to decide which method is the most appropriate and then base their conclusions on that method. Given that the authors are not aware of any studies that have determined the validity and reliability of GRADE, a qualitative assessment, and despite assessing different elements than the DAts, the authors would have defaulted to results from the DAts index given that it is a quantitative measure that has been shown to have excellent discriminatory metrics [16]. However, the authors note that additional research is needed on the discriminatory ability of the DAts index across different scenarios, something that is discussed in more detail in the Strengths and Potential Limitations section that follows.

The results of the treatment effects meta-analysis, a secondary purpose of the current study, yielded similar and positive results for both physical function and pain in the current study as well as comparable findings to those reported in the original guidelines statement [12]. Both the point estimate and 95% CI exceeded the MDC of 0.15 in the direction of benefit for both physical function and pain among RA patients. In addition, and while not conducted in the guidelines meta-analysis [12], support for the overall findings was reinforced when they remained statistically significant based on leave-one-out and outlier analysis for both physical function and pain. For pain, overall results were further supported by a lack of statistically significant between-study heterogeneity as well as small-study effects. In contrast, the statistically significant between-study heterogeneity and minor asymmetry suggestive of small-study effects for changes in physical function warrant more caution in the interpretation of the overall findings.

While not conducted in the original guidelines meta-analyses [12], the NNT based on the mean and 95% CI was laudable for both physical function and pain, with approximately 1.3 to 3.7 million physically inactive adults with RA worldwide improving their physical function and 1.3 to 3.8 decreasing their pain by exercising. In contrast, the 95% PI for both physical function and pain was wider and included the null, although the majority of the interval was on the side of benefit, with ~ 7 million people possibly improving their physical function and ~ 1 million seeing no benefit or a decrease. For pain, estimates were ~ 5.7 million people for decreases in pain and more than 700,000 for no change or increases. The results based on 95% PI provide one with a more accurate estimate regarding the true distribution of effects one might find in similar RA populations beyond the included studies and should be preferred clinically over 95% CI. Thus, while it is expected that a majority of physically inactive RA patients could improve their physical function and pain by exercising, a smaller number may see either no benefit or possible decreases in physical function and increases in pain as a result of exercise. Taken together, these findings, based on the mean as well as 95% CI and PI suggest that a substantial number of physically inactive adults with RA worldwide could improve their physical function and pain by exercising.

### Implications for research

The results of the current study suggest several areas for future research. First, and as previously mentioned, there is a need for additional, well-designed studies on the effects of exercise, regardless of type, on pain and physical function in adults with RA before true stability can be reached. These trials should be incorporated into future meta-analyses as well as any meta-analyses that might be conducted in conjunction with future guidelines statements. Second, there is a need for additional studies according to the type (aquatic, mind–body, etc.) and dose of exercise. For example, while no statistically significant between-group differences in either physical function or pain were observed between groups, the number of effect sizes in most groups was small, thereby affecting statistical power. Third, to optimize treatment guidelines, it would appear plausible to suggest that a need exists for a large component network meta-analysis (CNMA) [58] that includes both pharmacological and non-pharmacological interventions. Consolidating such evidence on different treatments into one CNMA would allow one to identify which interventions, combination of interventions, and components of these interventions (type, delivery method, etc.) would be best for improving physical function and pain, as well as other outcomes (fatigue, health-related quality-of-life, etc.) in adults with RA. Included in such an analysis should be potential harms. Finally, while the DAts index is currently limited to the IVhet [41] and Quality Effects (QE) models [59], it would appear reasonable to suggest that future methodological studies may be needed to compare the robustness of the DAts index based on models other than the IVhet and QE as well as the IVhet and QE models across diverse research fields and conditions.

### Implications for practice

There are several implications for practice. First, while the stability of exercise on changes in physical function and pain in adults with RA has not been firmly established, the current results suggest that, for a substantial number of adults with RA, exercise is a viable intervention for decreasing pain and increasing physical function. This may be especially true given the many other potential benefits of exercise as well as its excellent safety profile [60]. However, the ability to provide recommendations regarding the exact type or dose of exercise is currently not known and beyond the scope of the current study. Thus, until such information is available, including establishing whether true exercise-response variability actually exists [61], it would appear plausible to suggest that patients follow general physical activity guidelines, that is, at least 150–300 min of moderate intensity aerobic activity, 75–150 min of vigorous intensity aerobic activity, or some combination of both, spread throughout the week, as well as muscle strengthening activities for all major muscle groups of the body at least 2 days per week [62]. For aerobic activity (brisk walking, cycling, swimming), intensity may be monitored subjectively using the talk test approach whereby moderate intensity is where you can talk but not sing and vigorous intensity activity where you cannot sing and can only say a few words before needing to take a deep breath [63]. Consideration should also be given to the prevalence of kinesiophobia in many patients with RA, that is, an excessive, irrational fear of physical movement or activity stemming from a belief that movement will cause injury, re-injury, or increased pain [64, 65]. Potential strategies for dealing with kinesiophobia specific to RA patients have been described elsewhere [66].

### Implications for policy

From our perspective, there is a need for policies that support research on exercise and other treatment modalities for patients with RA. Doing so has the potential of benefiting not only the individual patient but also society as a whole, including the potential for reduced healthcare costs, especially for a treatment such as exercise. Second, given the lack of knowledge regarding exercise as well as competing demands of physicians and other healthcare providers, policies supporting the Exercise is Medicine (EIM) initiative would seem appropriate [67]. Led by the American College of Sports Medicine (ACSM), this initiative aims to make physical activity, including exercise, a standard part of healthcare by encouraging doctors to assess and prescribe exercise for patients. Importantly, it aims to connect patients with credentialed exercise professionals and resources. However, for this to occur, there is a need for policies that provide appropriate reimbursement for such.

### Strengths and potential limitations

From the authors’ viewpoint, the major strength of the current study was the first-time use of a novel quantitative approach, the DAts index [16], for determining the stability of exercise on pain and physical function in adults with RA. Secondary strengths included additional analyses not conducted in the original guidelines statement [12]. These included (1) calculation of 95% PI for determining what effect one might expect if they conducted their own RCT in similar populations of adults with RA [49], (2) leave-one-out analysis, (3) outlier analysis, (4) NNT analyses, and (5) gross estimates of the number of adults with RA worldwide who could improve their physical function and pain by exercising.

With respect to the DAts index, and from the investigative team’s perspective, there are several potential strengths of the DAts index. First, unlike an assessment instrument such as GRADE, which relies on “expert” judgment, it provides a quantitative metric to measure if the meta-analysis has reached “exit status”, that is, further studies will not change the conclusion. Second, the DAts index provides a concrete threshold for determining when a meta-analysis has reached sufficient stability to “exit” and conclude that no further research is needed on the topic. Third, the DAts index has shown highly effective discriminative ability in simulations and two real-world studies. In addition, one other recently published study has used the DAts index to determine conclusiveness [68]. Fourth, it provides a visual representation via a convergence plot that demonstrates how the DAts index behaves as new studies are added to a cumulative meta-analysis. Fifth, it has the potential to lower research waste by defining a clear exit point, and thus, prevents the continued accumulation of studies that are unlikely to change the overall conclusions.

In addition to strengths, there are several potential limitations of the DAts method. First, since this is a new method, it has not been tested under a variety of different scenarios. Thus, and while beyond the scope and purpose of the current meta-analysis and noted by the original study authors, additional testing of the DAts index itself under a variety of different scenarios [16] as well as against other existing quantitative and qualitative measures of conclusiveness is needed. Along those lines, a recent scoping review by Ban et al. [69] identified 62 different quantitative and qualitative methods to assess the conclusiveness of a meta-analysis. From the authors’ perspective, a large study comparing the strengths and limitations of each method against each other, including the DAts index, may provide important insights for selecting the best assessment method for a given scenario. Such a study may be especially worthwhile in determining if decisions regarding conclusiveness are method dependent. If there is method-dependency, one would then need to decide which method is the most plausible.

A second potential limitation is that the DAts index requires the use of cumulative meta-analysis in temporal sequence to evaluate stability [16]. Third, like most meta-analytic methods, its effectiveness may be influenced by the quality and heterogeneity of the studies included. Fourth, it may not be appropriate for meta-analyses with small, sparse datasets, including the subgroup analyses conducted in the current meta-analysis. Fifth, while the optimal cut point for the DAts aims to keep specificity above 90%, it may still require expert interpretation to confirm that “exiting” is appropriate in all contexts [16].

While a statistical comparison of the DAts index to the recently reported measures of conclusiveness that are currently available (*N* = 62, including the DAts index) was beyond both the purpose and scope of this study [69], the authors share their observations as it pertains to several other existing and commonly used frameworks. First, the GRADE assessment instrument is a subjective, qualitative evaluation of the certainty of evidence [13] while the DAts index is an objective, quantitative measure of the stability of the accumulated evidence [16]. Second, while both Trial Sequential Analysis (TSA) [70] and DAts indices include stopping rules to avoid premature or unnecessary updates, the DAts index measures “stability” rather than just “sufficiency” [16]. Third, while use of the Recursive Cumulative Meta-Analysis (RCM) procedure can demonstrate when an effect size is converging [71], it lacks a clear indicator of when to stop, i.e., “exit”, something the DAts index does provide [16]. Fourth, while both the DAts index and Optimal Information Size (OIS) [72] assess the robustness of a meta-analysis, the DAts index focuses on the stability of findings by adding studies whereas the OIS determines if the sample size is adequate. Thus, the DAts index is a quantitative measure of stability while the OIS relies on power calculations.

Beyond use of the DAts index, several other potential limitations should be acknowledged. First, since this was an aggregate data meta-analysis, the potential for aggregate data bias, i.e., ecological fallacy, exists. Second, like any meta-analysis, the study was limited by the data derived from the original studies. Third, while the additional moderator analyses yielded some results that differed from the overall treatment effect and exit meta-analysis results, the credibility of such should be considered very low given the small sample sizes in one or more groups as well as the possibility of chance findings given the large number of statistical tests conducted. In addition, these types of separate analyses may be misleading since they may be confounded by other factors, for example, those with RA who participate in aquatic exercise having higher baseline levels of pain than those who perform resistance exercise. Furthermore, the results of such analyses in an aggregate data meta-analysis may be misleading given that studies are not randomly assigned to moderators, and thus do not support causal inferences. Finally, and as previously noted, the focus of the current meta-analysis as well as original guidelines was on reaching general versus precision-based conclusions regarding a body of research [12, 18]. Ideally, some type of multiple meta-regression model after accounting for multicollinearity would probably be best for meta-analysis. However, this is rarely possible given (1) the small number of studies included in the vast majority of meta-analyses, including the current one, (2) the large number of moderators and/or mediators that would need to be considered, and (3) missing data for different variables from different studies.

Fourth, given the assumptions associated with NNT (constant relative effect across populations, baseline risk dependency, equal follow-up periods, homogeneity of effect size) [73, 74] and population estimates (target population mirrors the pooled cohort, no unobserved effect modifiers, generalizability, i.e., external validity) in an aggregate data meta-analysis, the current results should be interpreted cautiously.

Fifth, and while beyond the scope of the current study, the true benefit of exercise on pain and physical function in adults with RA, as well as the effects of any other treatments and outcomes, should probably be considered within a framework such as utility-based decision-making. This involves the assignment of numerical values (utilities) to outcomes, e.g., pain and physical function, that maximize decision-making while considering factors such as benefits, costs, harms, and personal preferences.

Sixth, while the authors used the conventional method for constructing prediction intervals [49], a statistic that is rarely reported in exercise intervention [75] as well as other intervention studies [49], additional and possibly more accurate methods such as bootstrap procedures have been proposed [76]. However, these also come with limitations [76]. Finally, extensions to prediction intervals that include the calculation of the expected proportion of comparable studies that resulted in clinically relevant benefit or harm have recently been proposed [77].

## Conclusions

The overall findings of the current study add to the American College of Rheumatology Guidelines assessment of “moderate” strength of evidence rating, suggesting that additional RCTs and subsequent meta-analyses are needed on the effects of exercise on physical function and pain in adults with RA before true stability/exit status can be reached.

## Supplementary Information

Below is the link to the electronic supplementary material.ESM1(DOCX 1.28 MB)

## Data Availability

All data associated with this study are available from the corresponding author upon reasonable request.

## Declarations

### PRISMA checklist

Supplementary file 13 contains the PRISMA checklist.

### Artificial intelligence

No artificial intelligence was used in this study.
