# Supplementary material for: Effects of exercise on physical function and pain in adults with rheumatoid arthritis: an exit meta-analysis of randomized controlled trials
Source: Clin Rheumatol. 2026 Jul 8;45(9):5165–77. doi: 10.1007/s10067-026-08274-w (PMC13526086; doi:10.1007/s10067-026-08274-w)
Supplement: Supplementary file 1 [file 10067_2026_8274_MOESM1_ESM.docx]

**SUPPLEMENTARY FILES**

**Supplementary file 1.** Stata code for DAts Index.

**Supplementary file 2.** Cochrane RoB2 risk of bias results.

**Supplementary file 3.** Forest plot figure, by exercise subgroup, for changes in physical function. Standardized mean difference effect size changes (Hedge’s g), adjusted for small study effects, for changes in physical function in adults with rheumatoid arthritis. The horizontal lines represent the lower and upper 95% confidence intervals from each study while the black squares represent the effect size from each study. The grey shading represents the weight applied to each study. The left and right sides of the diamonds represent the lower and upper 95% confidence intervals for the pooled effect size for each subgroup as well as the pooled effect size across all studies. The dashed vertical line represents the overall effect size across all studies while the solid vertical line represents the null (0).

**Supplementary file 4.** Doi plot and LFK index for small-study effects based on changes in physical function. The vertical axis represents normal quantiles (Z-scores) plotted again each studies standardized mean difference effect size (Hedge’s g) on the horizontal axis.

**Supplementary file 5.** Leave-one-out analysis for changes in physical function. Pooled standardized mean difference effect size changes (Hedges g) in physical function with each study deleted from the model once. The horizontal lines represent the pooled lower and upper 95% confidence intervals and the black square the pooled effect size with the study directly to the left deleted from the model. The grey shading represents the total weight of all other studies with the study directly to the left deleted from the model. The left and right dashed vertical lines represent the lower and upper 95% confidence intervals while the dashed vertical line in the middle represents the overall standardized mean difference effect size (Hedge’s g) with all studies pooled.

**Supplementary file 6.** Moderator and exit meta-analysis results table for changes in physical function.

**Supplementary file 7.** Forest plot, by exercise subgroup, for changes in pain. Standardized mean difference effect size changes (Hedge’s g), adjusted for small study effects, for changes in pain in adults with rheumatoid arthritis. The horizontal lines represent the lower and upper 95% confidence intervals from each study while the black squares represent the effect size from each study. The grey shading represents the weight applied to each study. The left and right sides of the diamonds represent the lower and upper 95% confidence intervals for the pooled effect size for each subgroup as well as the pooled effect size across all studies. The dashed vertical line represents the overall effect size across all studies while the solid vertical line represents the null (0).

**Supplementary file 8.** Doi plot and LFK index for small-study effects based on changes in pain. The vertical axis represents normal quantiles (Z-scores) plotted again each studies standardized mean difference effect size (Hedge’s g) on the horizontal axis.

**Supplementary file 9.** Leave-one-out analysis for changes in pain. Pooled standardized mean difference effect size changes (Hedges g) in pain with each study deleted from the model once. The horizontal lines represent the pooled lower and upper 95% confidence intervals and the black square the pooled effect size with the study directly to the left deleted from the model. The grey shading represents the total weight of all other studies with the study directly to the left deleted from the model. The left and right dashed vertical lines represent the lower and upper 95% confidence intervals while the dashed vertical line in the middle represents the overall standardized mean difference effect size (Hedge’s g) with all studies pooled.

**Supplementary file 10.** Moderator analyses table, including exit meta-analysis results, for changes in pain.

**Supplementary file 11**. Leave-one-out analysis for the Doi-Abdulmajeed trial stability index (DAts) for physical function.

**Supplementary file 12**. Leave-one-out analysis for the Doi-Abdulmajeed trial stability index (DAts) for pain.

**Supplementary file 13.** PRISMA Checklist.

**Supplementary file 1.** Stata code for DATS Index.

* exitma version 1.1 - 11 Sep 2024

* Authors: Luis Furuya-Kanamori (l.furuya@uq.edu.au), Suhail AR Doi, Jazeel AbdulMajeed

*v1.1 revised reference (in help file) and fig y-label

program define exitma, rclass

version 14

syntax varlist(min=2 max=4 numeric) [if] [in] ///

, SEQuence(varname) [, samplesize(varname)] ///

[, ivhet QE(varname numeric) noGraph]

tokenize `varlist'

preserve

marksample touse, novarlist

quietly keep if `touse'

************************

*Check required packages

foreach package in admetan {

capture which `package'

if _rc==111 ssc install `package'

}

************************

*Data input for analysis

*ES seES

if "`3'" == "" & "`4'" == "" {

display ""

display as text "Data input format ES seES assumed"

quietly{

gen __ES = `1'

gen __seES = `2'

}

*check entry error (negative SE and all positive ES)

sort `2'

if `2'[1] <= 0 {

di as error "{bf:`2'} cannot contain negative values"

exit 198

}

sort `1'

if `1'[1] > 0 {

di in red "verify {bf:`1'} is log transformed"

}

}

*ES lci uci

if "`3'" != "" & "`4'" == "" {

display ""

display as text "Data input format ES lci uci assumed"

quietly{

gen __ES = `1'

gen __lci = `2'

gen __uci = `3'

gen __seES = (__ES - __lci)/(invnormal(0.975))

}

*check entry error (all positive ES)

sort `1'

if `1'[1] > 0 {

di in red "verify {bf:`1'} is log transformed"

}

}

*event_treat noevent_treat event_ctrl noevent_ctrl

if "`3'" != "" & "`4'" != ""{

display ""

display as text "Data input format t_cases t_non-cases c_cases c_non-cases assumed"

quietly{

gen __a = `1'

gen __b = `2'

gen __c = `3'

gen __d = `4'

*cont correction

gen __continuity = 1 if __a==0 | __c==0

replace __a = __a+0.5 if __continuity ==1

replace __b = __b+0.5 if __continuity ==1

replace __c = __c+0.5 if __continuity ==1

replace __d = __d+0.5 if __continuity ==1

gen __ES = ln((__a*__d)/(__b*__c))

gen __seES = sqrt((1/__a)+(1/__b)+(1/__c)+(1/__d))

}

*check entry error (negative and non-integers)

sort `1'

if `1' <0{

di as error "{bf:`1'} cannot contain negative values"

exit 198

}

sort `2'

if `2'[1] <0{

di as error "{bf:`2'} cannot contain negative values"

exit 198

}

sort `3'

if `3'[1] <0{

di as error "{bf:`3'} cannot contain negative values"

exit 198

}

sort `4'

if `4'[1] <0{

di as error "{bf:`4'} cannot contain negative values"

exit 198

}

cap assert int(`1')==`1'

if _rc!=0{

di as error "{bf:`1'} contains non-integers"

exit _rc

}

cap assert int(`2')==`2'

if _rc!=0{

di as error "{bf:`2'} contains non-integers"

exit _rc

}

cap assert int(`3')==`3'

if _rc!=0{

di as error "{bf:`3'} contains non-integers"

exit _rc

}

cap assert int(`4')==`4'

if _rc!=0{

di as error "{bf:`4'} contains non-integers"

exit _rc

}

}

*************************

*Cumulative meta-analysis

gen __studyseq = `sequence'

sort __studyseq

gen __k_studies= __studyseq[_N]

local k_studies = __k_studies

if "`qe'"!="" & "`ivhet'"!="" {

display as error "specify only one model"

exit 198

}

if "`qe'"=="" {

quietly {

admetan __ES __seES, or ivhet cumulative nograph nowarn notable

qui gen __ma_es = _ES[_N] in 1

gen __se_ma_es = _seES[_N] in 1

}

display as text "IVhet model selected"

}

if "`qe'"!="" {

quietly {

admetan __ES __seES, or qe(`qe') cumulative nograph nowarn notable

qui gen __ma_es = _ES[_N] in 1

gen __se_ma_es = _seES[_N] in 1

}

display as text "QE model selected"

}

*****

*DAts

quietly {

gen __delta_c = _ES[_N]-_ES

gen __se_delta_c = sqrt(_seES^2 + (_seES[_N])^2)

gen __lower = 0 - 0.15

gen __upper = 0 + 0.15

gen __sample_size = .

if "`4'" != "" {

replace __sample_size = __a + __b + __c + __d

}

if "`4'" == "" {

cap assert `samplesize'!=.

if _rc {

display as error "specify samplesize"

exit 198

}

replace __sample_size = `samplesize'

}

gen __n = 0

replace __n = __sample_size in 1

replace __n = __sample_size[_n] + __n[_n-1] in 2/`k_studies'

gen __total_n = __n[_N]

gen __prop = __n/__total_n

summ __delta_c if __prop>=0.5, det

gen __dats = (r(Var)-0.002)*10 in 1

gen __k_studies50 = 1 if __prop>=0.5

egen __k_studies50max = count(__k_studies50)

local k_studies50max = __k_studies50max

*scalar

return scalar nstudy50 = __k_studies50max[1]

return scalar nstudy = __k_studies[1]

return scalar dats = __dats[1]

}

*display DAts

qui tostring __dats, gen(__dats_str) force

if __dats !=. {

qui gen ___dats_str1 = substr(__dats_str,1, strpos(__dats_str,".")+4)

local dats_str1 = ___dats_str1[1]

display ""

di as text "DAts index = " `dats_str1'

}

if __dats ==. {

di ""

di as text "DAts index cannot be computed, single study with >= 50% of the total participants"

}

*display study number

di as text "Total number of studies = " `k_studies'

di as text "Number of steps at or beyond 50% of participants = " `k_studies50max'

*****

*Plot

if "`graph'" != "nograph"{

twoway (rarea __lower __upper __prop, sort fcolor(gs10) fintensity(30) lcolor(white) cmissing(y)) ///

(connected __delta_c __prop, sort mcolor(navy) msymbol(circle) msize(large) lcolor(navy) yline(-.165 .165, lwidth(medthick) lpattern(dash) extend) xline(.5, lwidth(medthick) lpattern(dash) extend)), ///

ytitle(ΔCi) ytitle(, size(vlarge)) ylabel(, labsize(vlarge) angle(horizontal) labgap(small)) ///

xtitle(Cumulative proportion of participants) xtitle(, margin(medium) size(vlarge)) xlabel(0(.25)1, labsize(vlarge) labgap(small)) ///

legend(off) graphregion(fcolor(white)) ///

title(DAts = `dats_str1', size(medium) margin(medium))

}

restore

end

exit

**Supplementary file 2.** Cochrane RoB2 risk of bias results.

**Supplementary file 3.** Forest plot figure, by subgroup, for changes in physical function.

**Supplementary file 4.** Doi plot and LFK index for small-study effects based on changes in physical function.

**Supplementary file 5.** Leave-one-out analysis for changes in physical function.

| **Supplementary file 6.** Subgroup Results for Physical Function. | | | | | | | | | | | |
| --- | --- | --- | --- | --- | --- | --- | --- | --- | --- | --- | --- |
| Group | ES# | N | Hedges g  (95% CI) | z (p) | Q (p) | *I^2^* (95% CI) | Tau^2^ | Q_b_ (p) | 95% PI | DAts | Exit? |
| -AE | 9 | 925 | -0.35 (-0.56, -0.13) | -3.12 (0.002)* | 16.2 (0.04)** | 50.5 (0.0, 78.1 | 0.05 | 3.52 (0.32) | -0.92, 0.22 | 0.04 | P |
| -RT | 7 | 336 | -0.14 (-0.35, 0.08 | -1.24 (0.22) | 5.9 (0.43) | 0.0 (0.0, 57.8 | 0.00 |  | -0.35, 0.08 | -0.006 | Y |
| -AQ | 3 | 180 | -0.45 (-0.97, 0.08) | -1.66 (0.10) | 5.9 (0.05)** | 66.1 (0.0, 90.9) | 0.14 |  | -6.29, 5.40 | 0.106 | N |
| -MB | 1 | 46 | -0.59 (-1.19, -0.003 | -1.97 (0.05)* | NC | NC | NC |  | NC | NC | NC |
| -UC | 8 | 604 | -0.34 (-0.57, -0.11) | -2.90 (0.004)* | 9.78 (0.20) | 28.4 (0.0, 69.2) | 0.03 | 0.07 (0.79) | -0.83, 0.15 | -0.003 | Y |
| -OC | 12 | 883 | -0.30 (-0.50, -0.09 | -2.86 (0.004)* | 22.46 (0.02)** | 51.0 (0.0, 75.8) | 0.06 |  | -0.89, 0.30 | 0.054 | N |
| -HROB | 14 | 985 | -0.38 (-0.59, -0.17) | -3.6 (<0.001)* | 24.05 (0.03)** | 45.9 (0.0, 72.5) | 0.05 | 1.56 (0.21) | -0.93, 0.18 | 0.018 | P |
| -SCROB | 6 | 502 | -0.20, (-0.39, -0.001 | -1.97 (0.05)* | 5.72 (0.34) | 12.5 (0.0, 66.0) | 0.01 |  | -0.56, 0.17 | 0.00 | P |
| -AE & UC | 2 | 313 | -0.25 (-0.66, 0.17) | -1.16 (0.25) | 1.52 (0.22) | 34.2 (0.0, 86.9) | 0.04 | 0.38 (0.54) | NA | -0.009 | Y |
| -AE & OC | 7 | 612 | -0.40 (-0.66,-0.14 | -2.99 (0.003)* | 13.45 (0.04)** | 55.4 (0.0, 81.8) | 0.06 |  | -1.13, 0.33 | -0.01 | Y |
| -RT & UC | 4 | 181 | -0.27 (-0.57, 0.02) | -1.81 (0.07) | 3.0 (0.39) | 0.0 (0, 67.9) | 0.0 | 1.77 (0.18) | -0.57, 0.02 | -0.002 | Y |
| -RT & OC | 3 | 155 | -0.02 (-0.29, 0.34) | 0.13 (0.90) | 1.13 (0.57) | 0.0 (0.0, 53.2) | 0.0 |  | -0.2, 0.34 | 0.009 | P |
| -AQ & UC | 2 | 110 | -0.75 (-1.14, -0.36) | -3.80  (<0.001)* | 0.03 (0.86) | 0 (0.0, 0.0) | 0.0 | 5.87 (0.02)*\| | NC | NC | NC |
| -AQ & OC | 1 | 70 | 0.00 (-0.47, 0.47) | 0.00 (1.0) | NC | NC | NC |  | NC | NC | NC |
| -MB & UC | 1 | 46 | -0.59 (-1.19, -0,003) | -1.97 (0.05)* | NC | NC | NC | NC | NC | NC | NC |
| -MB & OC | NC | NC | NC | NC | NC | NC | NC |  | NC | NC | NC |
| -AE & HROB | 5 | 545 | -0.41 (-0.76, -0.07) | -2.33 (0.02)* | 9.88 (0.04)** | 59.5 (0.0, 86.2) | 0.07 | 0.54 (0.46) | -1.42, 0.59 | 0.069 | N |
| -AE & SCROB | 4 | 380 | -0.25 (-0.52, 0.03) | -1.73 (0.08) | 4.78 (0.19) | 37.2 (0.0, 80.1) | 0.03 |  | -1.19, 0.70 | 0.028 | P |
| -RT & HROB | 5 | 214 | -0.19 (-0.52, 0.14) | -1.11 (0.27) | 5.47 (0.24) | 26.9 (0.0, 73.9) | 0.04 | 0.31 (0.58) | -1.00, 0.63 | 0.006 | N |
| -RT & SCROB | 2 | 122 | -0.05 (-0.41, 0.31) | -0.27 (0.78) | 0.07 (0.80) | 0.0 (0.0, 0.0) | 0.0 |  | NC | -0.010 | Y |
| AQ & HROB | 3 | 180 | -0.45 (-0.97, 0.08) | -1.66 (0.10) | 5.90 (0.05)** | 66.1 (0.0, 90.9) | 0.14 | NC | -6.29, 5.40 | -0.106 | N |
| AQ & SCROB | NC | NC | NC | NC | NC | NC | NC |  | NC | NC | NC |
| MB & HROB | 1 | 46 | -0.59 (-1.19, -0.003) | -1.97 (0.05) | NC | NC | NC | NC | NC | NC | NC |
| MB & SCROB | NC | NC | NC | NC | NC | NC | NC |  | NC | NC | NC |
| Notes: ES#, number of effect sizes; N, Number of participants; Hedges g (95% CI), Hedges mean g and 95% confidence interval; z (p), z and alpha value for z; Q (p), Cochran’s Q statistic and alpha value for Q; *I^2^* (95% CI), I-squared and 95% confidence interval for I-squared; 95% PI, 95% prediction interval; Q_b_ (p), Between-group difference and alpha value; DAts, Doi-Abdulmajeed Trial Stability index; Exit, exit status for results, Y = Yes, N = No, P = Possibly; AE, Aerobic Exercise; RT, Resistance Training; AQ, Aquatic; MB, Mind-Body; UC, Usual Care; OC, Other Control Types; HROB, High Risk for Bias; SCROB, Some Concerns for Risk of Bias; NA, not calculable (lack of effect sizes); *, statistically significant (p ≤ 0.05); **, statistically significant (p ≤ 0.10); I, credibility considered “very low” based on ICEMAN criteria. | | | | | | | | | | | |

**Supplementary file 7.** Forest plot, by subgroup, for changes in pain.

**Supplementary file 8.** Doi plot and LFK index for small-study effects based on changes in pain.

**Supplementary file 9.** Leave-one-out analysis for changes in pain.

| **Supplementary file 10.** Subgroup results for changes in pain. | | | | | | | | | | | |
| --- | --- | --- | --- | --- | --- | --- | --- | --- | --- | --- | --- |
| Group | ES# | N | Hedges g  (95% CI) | z (p) | Q (p) | *I^2^* (95% CI) | Tau^2^ | 95% PI | Q_b_ (p) | DAts | Exit? |
| -AE | 4 | 488 | -0.37 (-0.76, 0.02) | -1.84 (0.07) | 10.76 (0.01)** | 72.1 (0.0, 91.2) | 0.10 | -2.00, 1.26 | 0.27 (0.98) | 0.0005 | P |
| -RT | 6 | 321 | -0.28 (-0.50, -0.05) | -2.44 (0.02)* | 3.54 (0.62) | 0.0 (0.0, 45.2) | 0 | -0.50, -0.05 |  | -0.015 | Y |
| -AQ | 3 | 143 | -0.31 (-0.85, 0.23) | -1.12 (0.26) | 4.56 (0.10) | 56.1 (0.0, 88.2) | 0.12 | -5.93, 5.32 |  | -0.002 | Y |
| -MB | 1 | 46 | -0.31 (-0.90, 0.27) | -1.1 (0.29) | NC | NC | NC | NC |  | NC | NC |
| -UC | 7 | 487 | -0.25 (-0.43, -0.06) | -2.54 (0.01)* | 6.18 (0.40) | 2.9 (0.0, 59.7) | 0.002 | -0.52, 0.03 | 0.96 (0.33) | -0.016 | Y |
| -OC | 7 | 511 | -0.40 (-0.66, -0.15) | -3.13 (0.002)* | 11.66 (0.07)** | 48.5 (0.0, 78.7) | 0.06 | -1.09, 0.29 |  | -0.008 | Y |
| -HROB | 11 | 785 | -0.37 (-0.58, -0.16) | -3.40 (0.001)* | 17.59 (0.06)** | 43.1 (0.0, 72.7) | 0.05 | -0.92, 0.18 | 1.19 (0.28) | 0.016 | P |
| -SCROB | 3 | 213 | -0.17 (-0.45, 0.11) | -1.21 (0.23) | 0.22 (0.90) | 0.0 (0.0, 0.0) | 0.00 | -0.45, 0.11 |  | -0.019 | Y |
| -AE & UC | 1 | 220 | -0.11 (-0.29, 0.17) | -0.78 (0.44) | NC | NC | NC | NC | 3.53 (0.06) | NC | NC |
| -AE & OC | 3 | 268 | -0.58 (-0.98, -0.18) | 2.85 (0.004)* | 4.89 (0.09)** | 59.1 (0.0, 88.9) | 0.07 | -4.87, 3.71 |  | -0.158 | N |
| -RT & UC | 4 | 194 | -0.24 (-0.52, 0.04) | -1.66 (0.10) | 1.78 (0.62) | 0.0 (0.0, 46.5) | 0 | -0.52, 0.04 | 0.11 (0.75) | -0.008 | Y |
| -RT & OC | 2 | 127 | -0.33 (-0.78, 0.12) | -1.44 (0.15) | 1.62 (0.20) | 38.2 (0.0, 87.7) | 0.04 | NC |  | 0.219 | N |
| -AQ & UC | 2 | 73 | -0.64 (-1.11, -0.16) | -2.63 (0.008)* | 0.91 (0.34) | 0.0 (0.0, 78.1) | 0 | NC | 3.65 (0.06) | NC | NC |
| -AQ & OC | 1 | 70 | 0.01 (-0.46, 0.48) | 0.05 (0.96) | NC | NC | NC | NC |  | NC | NC |
| -MB & UC | NC | NC | NC | NC | NC | NC | NC | NC | NC | NC | NC |
| -MB & OC | 1 | 46 | -0.31 (-0.90, 0.27) | -1.06 (0.29) | NC | NC | NC | NC |  | NC | NC |
| -AE & HROB | 3 | 400 | -0.42 (-0.92, 0.09) | -1.60 (0.11) | 9.67 (0.008)** | 79.3 (0.0, 94.5) | 0.14 | -6.21, 5.38 | 0.59 (0.44) | 0.222 | N |
| -AE & SCROB | 1 | 88 | -0.15 (-0.61, 0.31) | -0.63 (0.53) | NC | NC | NC | NC |  | NC | NC |
| -RT & HROB | 4 | 196 | -0.33 (-0.62, -0.05) | -2.30 (0.02)* | 2.94 (0.40) | 0.0 (0.0, 67.3) | 0.0 | -0.62, -0.05 | 0.40 (0.53) | 0.002 | P |
| -RT & SCROB | 2 | 125 | -0.19 (-0.54, 0.17) | -1.04 (0.30) | 0.20 (0.65) | 0.0 (0.0, 0.8) | 0 | NC |  | 0.009 | P |
| AQ & HROB | 3 | 143 | -0.31 (-0.85, 0.23) | -1.12 (0.26) | 4.56 (0.10) | 56.1 (0.0, 88.2) | 0.12 | -5.98, 5.36 | NC | -0.0002 | Y |
| AQ & SCROB | NC | NC | NC | NC | NC | NC | NC | NC |  | NC | NC |
| MB & HROB | 1 | 46 | -0.31 (-0.90, 0.27) | -1.06 (0.29) | NC | NC | NC | NC | NC | NC | NC |
| MB & SCROB | NC | NC | NC | NC | NC | NC | NC | NC |  | NC | NC |
| Notes: ES#, number of effect sizes; N, Number of participants; Hedges g (95% CI), Hedges mean g and 95% confidence interval; z (p), z and alpha value for z; Q (p), Cochran’s Q statistic and alpha value for Q; *I^2^* (95% CI), I-squared and 95% confidence interval for I-squared; 95% PI, 95% prediction interval; Q_b_ (p), Between-group difference and alpha value; DAts, Doi-Abdulmajeed Trial Stability index; Exit?, exit status for results, Y = Yes, N = No, P = Possibly; AE, Aerobic Exercise; RT, Resistance Training; AQ, Aquatic; MB, Mind-Body; UC, Usual Care; OC, Other Control Types; HROB, High Risk for Bias; SCROB, Some Concerns for Risk of Bias; NA, not calculable (lack of effect sizes); *, statistically significant (p ≤ 0.05); **, statistically significant (p ≤ 0.10). | | | | | | | | | | | |

| **Supplementary file 11.** Leave-one-out analysis results for physical function and exit status. | | |
| --- | --- | --- |
| Excluded Study | DAtS | Exit? |
| da Silva et al., 2013 | -0.0063 | Y |
| Breedland et al., 2011 | 0.0032 | P |
| Siqueira et al., 2017 | 0.0033 | P |
| Lemmey et al., 2009 | 0.005 | P |
| de Jong et al., 2003 | 0.0061 | P |
| Katz et al., 2017 | 0.0061 | P |
| Durcan et al., 2014 | 0.0067 | P |
| Hall et al., 1996 | 0.0071 | P |
| Hakkinen et al., 2001 | 0.0071 | P |
| Westby et al., 2000 | 0.0081 | P |
| Hakkinen et al., 1999 | 0.0084 | P |
| Garcia-Morales et al. 2020 | 0.009 | P |
| Puksic et al., 2021 | 0.0095 | P |
| Komatireddy et al., 1997 | 0.0097 | P |
| Siqueira et al., 2017 | 0.0098 | P |
| Hurkmans et al., 2010 | 0.0118 | P |
| Bilberg et al., 2005 | 0.0129 | P |
| Rodrigues et al., 2020 | 0.013 | P |
| VanDenBerg et al., 2006 | 0.0134 | P |
| Lourenzi et al., 2017 | 0.0178 | P |
| Notes: DAts, Doi-Abdulmajeed Trial Stability index; Exit?, exit status for results, Y = Yes, N = No, P = Possibly; Results ordered from smallest to largest. | | |
|  | | |

| **Supplementary file 12.** Leave-one-out analysis results for pain and exit status. | | |
| --- | --- | --- |
| Excluded Study | DAtS | Exit? |
| Hall et al., 1996 | -0.0155 | Y |
| Komatireddy et al., 1997 | -0.015 | Y |
| Hakkinen et al., 2001 | -0.0149 | Y |
| Hakkinen et al., 1999 | -0.0143 | Y |
| da Silva et al., 2013 | -0.012 | Y |
| Neuberger et al., 2007 | -0.0119 | Y |
| Durcan et al., 2014 | -0.009 | Y |
| Siqueira et al., 2017 | 0.0018 | P |
| Puksic et al., 2021 | 0.0054 | P |
| Lourenzi et al., 2017 | 0.0058 | P |
| Rodrigues et al., 2020 | 0.0059 | P |
| Katz et al., 2017 | 0.0063 | P |
| Bilberg et al., 2005 | 0.0074 | P |
| Rintala et al., 2005 | 0.0088 | P |
| Notes: DAts, Doi-Abdulmajeed Trial Stability index; Exit?, exit status for results, Y = Yes, N = No, P = Possibly; Results ordered from smallest to largest. | | |

| **Section and Topic** | **Item #** | **Checklist item** | **Location where item is reported** |
| --- | --- | --- | --- |
| **TITLE** | | |  |
| Title | 1 | Identify the report as a systematic review. | Page 1 |
| **ABSTRACT** | | |  |
| Abstract | 2 | See the PRISMA 2020 for Abstracts checklist. | Page 2 |
| **INTRODUCTION** | | |  |
| Rationale | 3 | Describe the rationale for the review in the context of existing knowledge. | Pages 3 and 4 |
| Objectives | 4 | Provide an explicit statement of the objective(s) or question(s) the review addresses. | Page 4 |
| **METHODS** | | |  |
| Eligibility criteria | 5 | Specify the inclusion and exclusion criteria for the review and how studies were grouped for the syntheses. | Page 4 and 5 |
| Information sources | 6 | Specify all databases, registers, websites, organisations, reference lists and other sources searched or consulted to identify studies. Specify the date when each source was last searched or consulted. | Page 5 |
| Search strategy | 7 | Present the full search strategies for all databases, registers and websites, including any filters and limits used. | Not applicable |
| Selection process | 8 | Specify the methods used to decide whether a study met the inclusion criteria of the review, including how many reviewers screened each record and each report retrieved, whether they worked independently, and if applicable, details of automation tools used in the process. | Page 5 |
| Data collection process | 9 | Specify the methods used to collect data from reports, including how many reviewers collected data from each report, whether they worked independently, any processes for obtaining or confirming data from study investigators, and if applicable, details of automation tools used in the process. | Pages 5 and 6 |
| Data items | 10a | List and define all outcomes for which data were sought. Specify whether all results that were compatible with each outcome domain in each study were sought (e.g. for all measures, time points, analyses), and if not, the methods used to decide which results to collect. | Page 6 |
|  | 10b | List and define all other variables for which data were sought (e.g. participant and intervention characteristics, funding sources). Describe any assumptions made about any missing or unclear information. | Pages 6 and 7 |
| Study risk of bias assessment | 11 | Specify the methods used to assess risk of bias in the included studies, including details of the tool(s) used, how many reviewers assessed each study and whether they worked independently, and if applicable, details of automation tools used in the process. | Pages 8 and 9 |
| Effect measures | 12 | Specify for each outcome the effect measure(s) (e.g. risk ratio, mean difference) used in the synthesis or presentation of results. | Page 6 |
| Synthesis methods | 13a | Describe the processes used to decide which studies were eligible for each synthesis (e.g. tabulating the study intervention characteristics and comparing against the planned groups for each synthesis (item #5)). | Page 5 |
|  | 13b | Describe any methods required to prepare the data for presentation or synthesis, such as handling of missing summary statistics, or data conversions. | Page 8 and 9 |
|  | 13c | Describe any methods used to tabulate or visually display results of individual studies and syntheses. | Pages 9 and 10 |
|  | 13d | Describe any methods used to synthesize results and provide a rationale for the choice(s). If meta-analysis was performed, describe the model(s), method(s) to identify the presence and extent of statistical heterogeneity, and software package(s) used. | Pages 6-7, 11, Supplementary file 1 |
|  | 13e | Describe any methods used to explore possible causes of heterogeneity among study results (e.g. subgroup analysis, meta-regression). | Pages 7-8, 10-11 |
|  | 13f | Describe any sensitivity analyses conducted to assess robustness of the synthesized results. | Page 7 |
| Reporting bias assessment | 14 | Describe any methods used to assess risk of bias due to missing results in a synthesis (arising from reporting biases). | Pages 8-9 |
| Certainty assessment | 15 | Describe any methods used to assess certainty (or confidence) in the body of evidence for an outcome. | Pages 9-11 |
| **RESULTS** | | |  |
| Study selection | 16a | Describe the results of the search and selection process, from the number of records identified in the search to the number of studies included in the review, ideally using a flow diagram. | Pages 11-12 |
|  | 16b | Cite studies that might appear to meet the inclusion criteria, but which were excluded, and explain why they were excluded. | Not applicable |
| Study characteristics | 17 | Cite each included study and present its characteristics. | Not applicable |
| Risk of bias in studies | 18 | Present assessments of risk of bias for each included study. | Supplementary file 2 |
| Results of individual studies | 19 | For all outcomes, present, for each study: (a) summary statistics for each group (where appropriate) and (b) an effect estimate and its precision (e.g. confidence/credible interval), ideally using structured tables or plots. | Table 1, Figures 1 and 2, Supplementary files 3 and 7 |
| Results of syntheses | 20a | For each synthesis, briefly summarise the characteristics and risk of bias among contributing studies. | Pages 11-12, Supplementary file 2 |
|  | 20b | Present results of all statistical syntheses conducted. If meta-analysis was done, present for each the summary estimate and its precision (e.g. confidence/credible interval) and measures of statistical heterogeneity. If comparing groups, describe the direction of the effect. | Pages 12-16 Table 1, Supplementary files 3, 6-7, 10 |
|  | 20c | Present results of all investigations of possible causes of heterogeneity among study results. | Pages 12-14, Supplementary 3, 6, 7, 10 |
|  | 20d | Present results of all sensitivity analyses conducted to assess the robustness of the synthesized results. | Pages 12-16, Supplementary files 3, 5-7, 9- 12 |
| Reporting biases | 21 | Present assessments of risk of bias due to missing results (arising from reporting biases) for each synthesis assessed. | Pages 11-14, Supplementary files 2, 4, 8 |
| Certainty of evidence | 22 | Present assessments of certainty (or confidence) in the body of evidence for each outcome assessed. | Pages 14-16, Figures 3 and 4 |
| **DISCUSSION** | | |  |
| Discussion | 23a | Provide a general interpretation of the results in the context of other evidence. | Pages 16-18 |
|  | 23b | Discuss any limitations of the evidence included in the review. | Pages 21-25 |
|  | 23c | Discuss any limitations of the review processes used. | Pages 21-25 |
|  | 23d | Discuss implications of the results for practice, policy, and future research. | Pages 18-21 |
| **OTHER INFORMATION** | | |  |
| Registration and protocol | 24a | Provide registration information for the review, including register name and registration number, or state that the review was not registered. | Page 4 |
|  | 24b | Indicate where the review protocol can be accessed, or state that a protocol was not prepared. | Page 4 |
|  | 24c | Describe and explain any amendments to information provided at registration or in the protocol. | Pages 8-9, 11 |
| Support | 25 | Describe sources of financial or non-financial support for the review, and the role of the funders or sponsors in the review. | Pages 6, 26 |
| Competing interests | 26 | Declare any competing interests of review authors. | Pages 6, 26 |
| Availability of data, code and other materials | 27 | Report which of the following are publicly available and where they can be found: template data collection forms; data extracted from included studies; data used for all analyses; analytic code; any other materials used in the review. | Page 26 |

*From:* Page MJ, McKenzie JE, Bossuyt PM, Boutron I, Hoffmann TC, Mulrow CD, et al. The PRISMA 2020 statement: an updated guideline for reporting systematic reviews. BMJ 2021;372:n71. doi: 10.1136/bmj.n71. This work is licensed under CC BY 4.0. To view a copy of this license, visit <https://creativecommons.org/licenses/by/4.0/>
